# Deep Learning Approaches for Automatic Quality Assurance of Magnetic Resonance Images Using ACR Phantom

**DOI:** 10.1186/s12880-023-01157-5

**Published:** 2023-11-29

**Authors:** Tarraf Torfeh, Souha Aouadi, SA Yoganathan, Satheesh Paloor, Rabih Hammoud, Noora Al-Hammadi

**Affiliations:** Department of Radiation Oncology, National Center for Cancer Care & Research (NCCCR), Hamad Medical Corporation, Doha, Qatar

**Keywords:** MRI, Deep learning, Quality control

## Abstract

**Background:**

In recent years, there has been a growing trend towards utilizing Artificial Intelligence (AI) and machine learning techniques in medical imaging, including for the purpose of automating quality assurance. In this research, we aimed to develop and evaluate various deep learning-based approaches for automatic quality assurance of Magnetic Resonance (MR) images using the American College of Radiology (ACR) standards.

**Methods:**

The study involved the development, optimization, and testing of custom convolutional neural network (CNN) models. Additionally, popular pre-trained models such as VGG16, VGG19, ResNet50, InceptionV3, EfficientNetB0, and EfficientNetB5 were trained and tested. The use of pre-trained models, particularly those trained on the ImageNet dataset, for transfer learning was also explored. Two-class classification models were employed for assessing spatial resolution and geometric distortion, while an approach classifying the image into 10 classes representing the number of visible spokes was used for the low contrast.

**Results:**

Our results showed that deep learning-based methods can be effectively used for MR image quality assurance and can improve the performance of these models. The low contrast test was one of the most challenging tests within the ACR phantom.

**Conclusions:**

Overall, for geometric distortion and spatial resolution, all of the deep learning models tested produced prediction accuracy of 80% or higher. The study also revealed that training the models from scratch performed slightly better compared to transfer learning. For the low contrast, our investigation emphasized the adaptability and potential of deep learning models. The custom CNN models excelled in predicting the number of visible spokes, achieving commendable accuracy, recall, precision, and F1 scores.

## Background

Magnetic Resonance Imaging (MRI), due to technological advances, represents now an important imaging modality used for several clinical application such as diagnostic and guidance of localized therapies such as surgery, Brachytherapy, Radiation Therapy (RT) and High Intensity Focused Ultrasound (HI-FU) therapy [1–3]. MRI provides excellent soft tissue contrast and resolution as well as functional imaging capabilities, allowing for the spatial and physiological characterization of disease.

The premise of MR based clinical applications is that a successful diagnostic and therapeutic outcomes relies on accurate localization and good spatial resolution and contrast.

An essential pre-requisite for any image Guided (IG) clinical application is the comprehensive quality assurance of the imaging modality. As such, benchmarking and periodic assessment of image quality, spatial fidelity, geometric precision, and laser/ couch accuracy are critical.

For the image quality module, several phantoms and tests have been designed and implemented. The American College of Radiology (ACR) phantom and associated tests are the most widely used in MRI since the tests and metrics are standardized and widely accepted as they follow the recommendations outlined in the ACR and American Association Physicists in Medicine (AAPM) reports surveyed [4, 5].

The ACR phantom guidance tests are commonly adopted as a routine clinical QC and are traditionally conducted by manual methods. Manual tests consist of multiple labor-intensive measurements (slice thickness, slice location, contrast, and geometric distortion). Results obtained depend on user defined windowing levels and the guidance definitions of leveling and windowing are approximate and subjective. Slice selection for specific tests is also user dependent.

Several approaches for the development of software tools capable of automating MRI quality control procedure using the ACR phantom are available [6–12]. Most of these tools use conventional image processing methods [6–10] which rely on techniques from computer vision such as edge detection, segmentation, and feature extraction, to process images and extract useful information from them.

In recent years, there has been a growing trend towards the use of Artificial Intelligence (AI) and machine learning techniques in medical imaging [13–16], particularly MRI. One approach is to use AI algorithms to analyze MRI scans and identify patterns that are associated with specific diseases or conditions [17, 18]. AI algorithms can also be used for MR image reconstruction, denoising and 4D management [19, 20]. Another way that AI is being used in MRI is to automate the process of image interpretation, the low contrast test, in particular, which is considered one of the most challenging tests within the ACR phantom as it includes 30 regions with varying levels of visibility. These regions must be accurately classified as either visible or not visible. As such, two automatic approaches to evaluate the MR ACR low contrast resolution have been presented by Ramos et al. [11, 12]. The first one [11] uses machine learning models while the second approach [12] uses deep learning models. In both approaches features are first extracted from the image and used as an input to the AI models. In addition to evaluating MRI quality using phantoms, there are many automatic image quality assessment works for clinical MRIs that are more complex and use deep learning approaches [21, 22]. These studies demonstrate the growing interest in and advancements of AI for clinical MRIs.

This study investigates the use of deep learning for the automatic assessment of geometric distortion, spatial resolution, and low contrast in MRI images We chose to focus on low contrast because it is the most challenging parameter to assess. In addition to low contrast, we expand our exploration to encompass geometric distortion and spatial resolution tests, demonstrating the potential of deep learning techniques to revolutionize the assessment of diverse ACR quality control parameters. We first developed and tested convolutional neural network (CNN) models from scratch. Also, several models, including VGG16, VGG19, Resnet50, InceptionV3, EfficientNetB0 and EfficientNetB5 [23–26] were trained from scratch. The impact of the batch size on the results is also tested. We also explored the use of pretrained models, such as those trained on the ImageNet dataset, for transfer learning. This allowed us to leverage the knowledge gained from these models to improve the performance of our MR image quality assurance model. The methodologies presented in this paper distinguish themselves from their predecessors by a notable characteristic: they have the capability to process the entirety of MR images without necessitating the extraction of specific image features.

We used two-classes classification models for spatial resolution and geometric distortion. For the low contrast test, we used an approach classifying the image into 10 classes representing the number of spokes visible in the image.

## Methods

### Dataset

Our dataset includes images of 256 × 256 pixels^2^ issued from 120 acquisition of the ACR phantom. MRI image acquisition was performed on a GE 1.5TMRI-SIM,450 W unit commissioned for RT planning.

#### Spatial resolution and geometric distortion

**Fig. 1:**
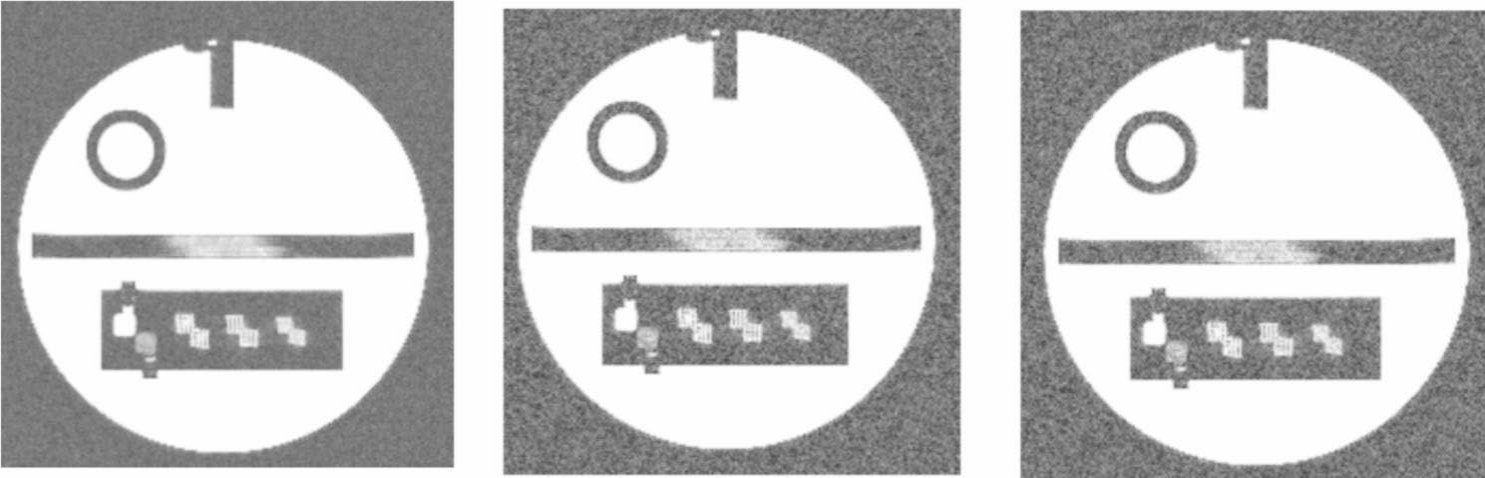
Noise simulation. (**A**) original MR image of ACR phantom. (**B**) image with noise using gaussian distribution with SD = 12. (**C**) image with noise using gaussian distribution with SD = 16

The spatial resolution test evaluates the scanner’s capability of resolving small objects when the contrast-to-noise ratio is high enough that it does not limit this ability. This test is based on a slice of the phantom showing three small grid structures as shown in Fig. 1.

The Geometric distortion test is performed to assess whether geometric distortion has occurred during the scan process. Geometric distortion test involves measurements on sagittal localizer and on axial slices as shown in Fig. 1.

To increase the amount of training data and to address the issue of class imbalance within the dataset, images for the spatial resolution and geometric distortion were degraded by simulating noise and blur. This approach augments the training data, thereby ensuring a balanced representation of both pass and fail classes.

The Gaussian distribution function is used to simulate noise, and the standard deviation of the probability distribution function is varied from 1 to 16 (Fig. 1). The choice of Gaussian noise for simulating noise in MRI was motivated by its close approximation to the real noise distribution observed in medical image acquisitions, including certain physics-related aspects of MR image acquisition. Moreover, Gaussian noise is easy to generate, which makes it a practical choice for simulating noise and augmenting the dataset.

**Fig. 2:**
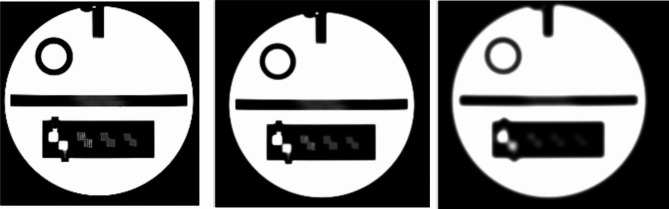
Blur simulation. (**A**) original MR image of the ACR phantom. (**B**) image with blur using PSF of FWHM = 3 mm. (**C**) image with blur using a PSF of 6 mm

Blur is simulated by convolving the images with a two-dimensional Gaussian function, known as a Point Spread Function (PSF), with the Full Width at Half Maximum varying from 1 to 16 mm (Fig. 2). As such, a total of around 600 images were generated for spatial resolution and geometric distortion assessments. Out of these, 240 were real MRI images, and the remaining images were simulated.

At the end, the dataset for these tests consisted of 600 labeled slices with 1 indicating that the test has passed and 0 indicating that it has failed. These slices were randomly split into three sets: training data (499 images), validation data (63 images), and testing data (63 images).

To increase the size of the training set and make the model more robust to variations in the data, data augmentation was applied to the original training images by performing random rotations of ± 7 degrees and translations of ± 2 mm.

The model’s output is a prediction number that ranges from 0 to 1, where a value closer to 1 indicates a higher probability of parameter to pass. The loss function adopted for training is “binary Cross-entropy " and the optimizer used is ADAM (Adaptive Moment Estimation).

**Fig. 3:**
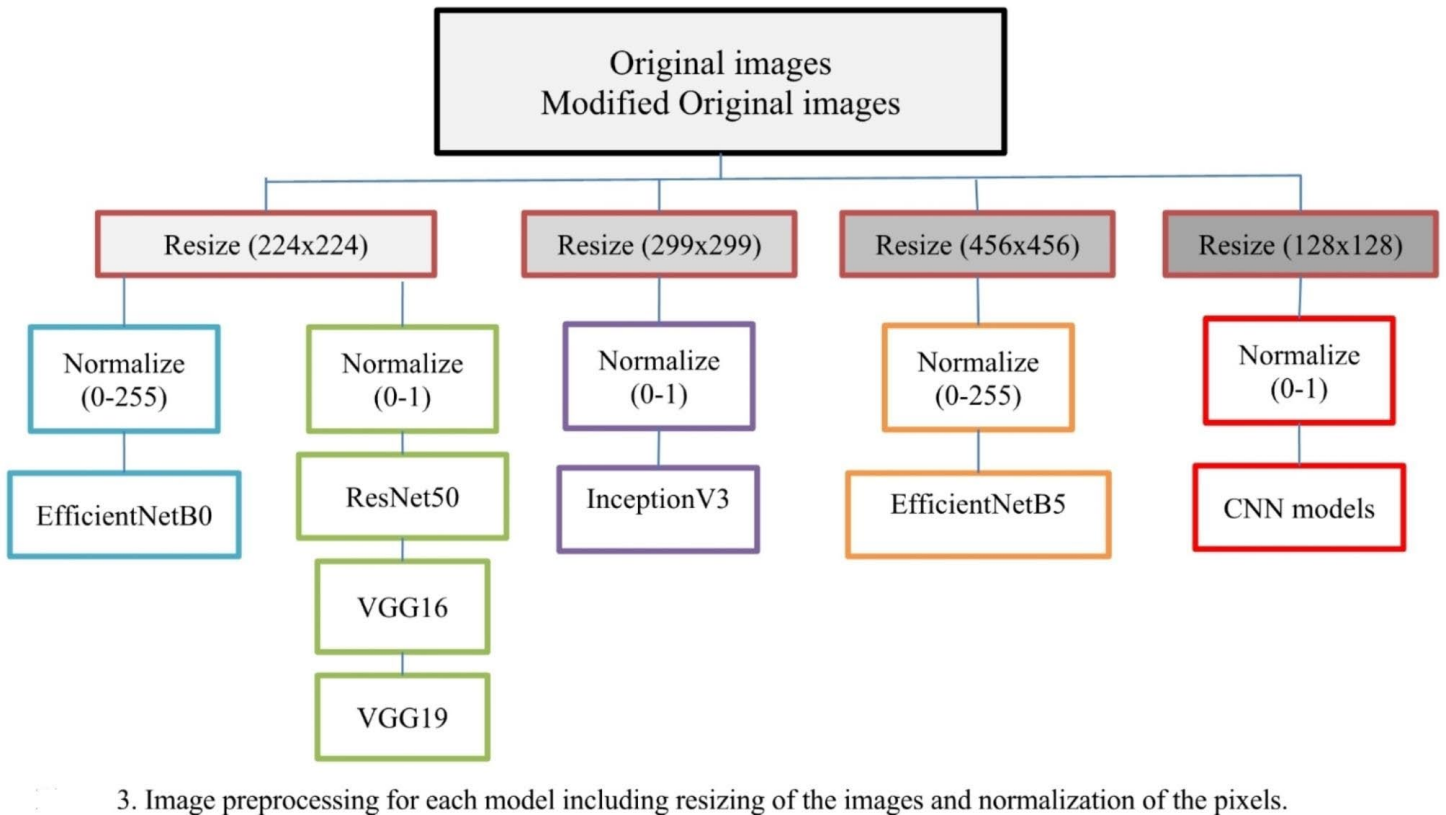
Image preprocessing for each model including resizing of the images and normalization of the pixels

Before training the models, it is essential to preprocess the images to ensure that they are suitable for the network to learn from as show in Fig. 3. One of the preprocessing steps involves normalizing the intensities of the pixels in the images.

For the EfficientNetB0 and EfficientNetB5 models, the intensities of the pixels were normalized to the range of 0 to 255. On the other hand, for the VGG16, VGG19, ResNet50, and InceptionV3 models, the intensities of the pixels were normalized to a range of 0 to 1. This range was chosen because it is the standard range used in these deep learning models.

Another important preprocessing step is resizing the images to a specific size. For ResNet50, VGG16, VGG19, and EfficientNetB0, the images were resized to 224 × 224 pixels. However, for EfficientNetB5 and InceptionV3, the images were resized to 456 × 456 and 299 × 299 pixels, respectively. This resizing step ensures that all images have the same size, allowing the model to process the data efficiently. Figure 3 illustrates the preprocessing procedure, which encompasses image resizing and standardization.

For the developed CNN models, intensities of the pixels were normalized to a range of 0 to 1 and images were resized to 256 × 256 pixels.

#### Low contrast

**Fig. 4:**
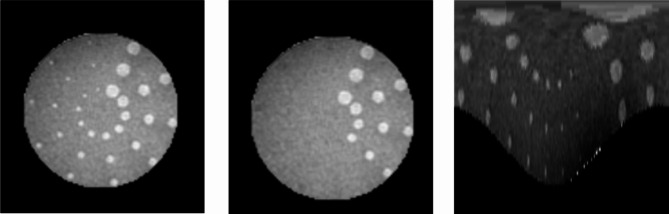
**A**) original MR image for the low contrast. **B**) image with 6 spokes masked. **C**) image with pixels transformed from cartesian to polar coordinates

The low-contrast objects appear on four slices of the ACR MR images. In each slice the low-contrast objects appear as rows of small disks, with the rows radiating from the center of a circle like spokes in a wheel as shown in Fig. 4. Low contrast test measures the number of spokes in each slice for which all holes is visible.

To increase the training data, we devised a novel approach wherein new images were generated through the masking of one or more spokes. Consequently, a single image could be utilized to create 10 distinct images, each featuring a specific number of visible spokes, as illustrated in Fig. 4. Additionally, circular cropping was applied to the images to eliminate external pixels, further enhancing the quality of the visual representations.

The dataset used to train, test and validate the models for the low contrast parameter consisted of 1000 slices. Every spoke in the images is classified as visible or not visible. These slices were randomly split into three sets: training data (800 images), validation data (100 images), and testing data (100 images). To increase the size of the training set and make the model more robust to variations in the data, data augmentation was applied to the original training images by performing random rotations of ± 7 degrees and translations of ± 2 mm.

To enhance the performance of our models, we conducted a series of preprocessing experiments on the images involving Fourier transform, convolution, and other techniques. Through thorough exploration, we found that transforming pixels from Cartesian to polar coordinates yielded the most effective results.

The utilization of polar image transformations within neural networks has been previously investigated in the literature [27, 28] especially when segmenting multiple elliptical objects. The key advantage lies in the ability of polar image transformations to decouple the segmentation and localization tasks. In our specific research context, where our objective is the detection of circular objects, this transformation has proven to be highly effective. In comparison to alternative preprocessing methods, which yielded accuracy rates below 20%, the utilization of the Cartesian to polar transformation consistently yielded higher accuracy levels.

As a result, the ultimate step in our methodology revolves around this transformative process as shown in Fig. 4. The conversion to polar coordinates demonstrated superior performance in enhancing the accuracy of the predicting models leading us to adopt this technique as the ultimate step in our analysis.

The pixel intensities and image size were normalized following the same procedures as applied to address geometric distortion and spatial resolution.

### Artificial intelligence models

**Fig. 5:**
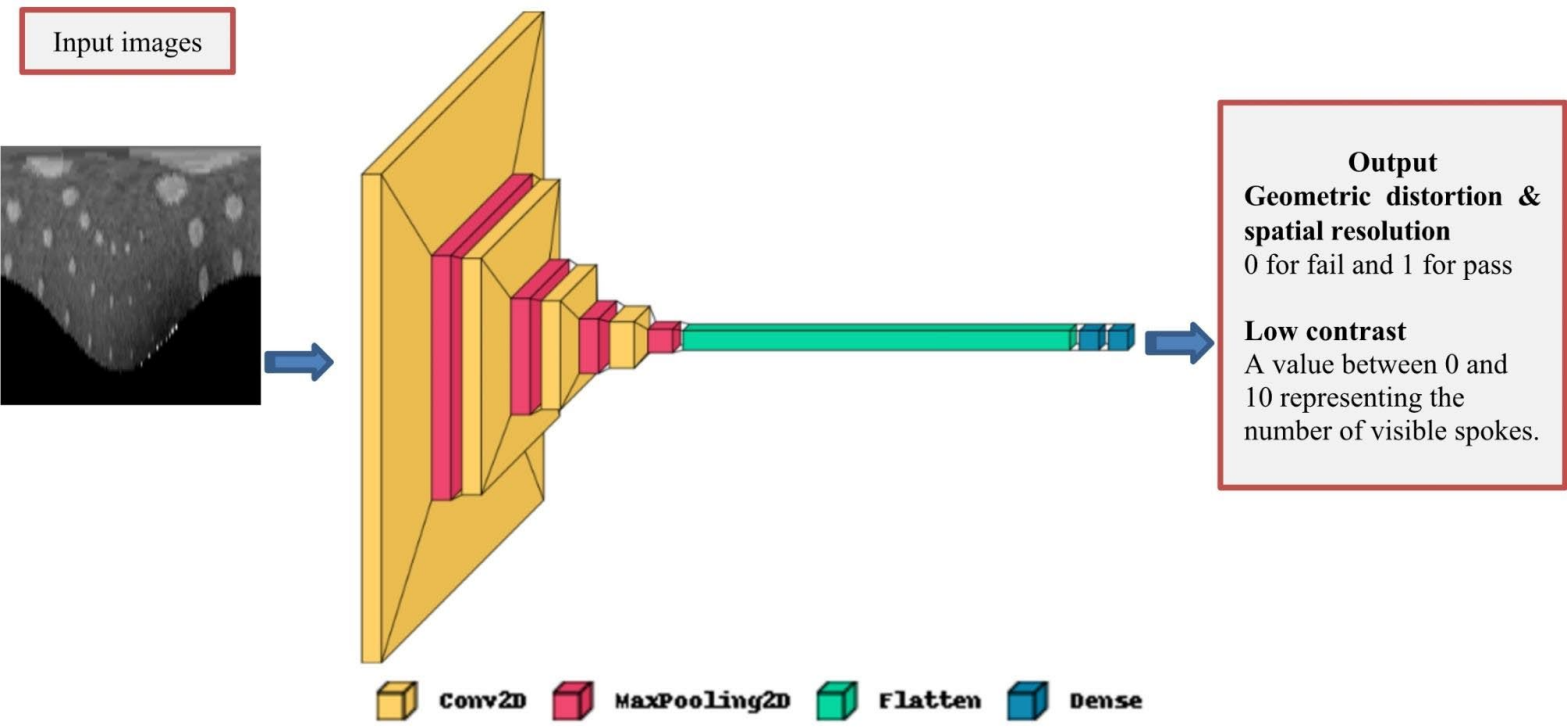
First CNN model containing four convolutional layers, four max pooling layers, a flattening layer, and two dense layers

Our first model (Fig. 5) consists of four convolutional layers with adjustable filter sizes, four max pooling layers for down sampling, a flattening layer, and two dense layers with adjustable units. The model is optimized with the Adam optimizer and categorical cross-entropy loss function.

For the second model, we have added two additional convolutional layers and an additional pooling layer after each convolutional layer. This increases the depth of the model, allowing it to capture more complex features and patterns in the images.

Furthermore, a variety of different architectures were implemented and evaluated including VGG16, VGG19, Resenet50, InceptionV3, EfficinetNetB0 and EfficientNetB5.

These deep learning models have been widely used for tasks in the medical imaging field, as well as for image classification tasks more generally. Furthermore, for the deep learning models, we used a transfer learning approach that involves using a pre-trained model already trained on a large dataset (ImageNet in our case) as a starting point. The top layer of these models has been replaced with a new classification head that is tailored to our specific task (binary or multi-class classification task). This approach allows us to leverage the features learned by the base model and apply them to the new task, while still allowing the model to learn task-specific features through the new top layer.

Two scenarios are being considered. In the first scenario, the transfer learning scenario, the model’s weights, except for the classifier, are set as non-trainable. This means that during the training process, only the dense layer weights will be updated and optimized, while the other weights in the model will remain fixed. This approach can be useful when fine-tuning a pre-trained model, as it allows the classifier to adapt to the new data without drastically changing the feature extraction capabilities of the model.

In the second scenario, all the model’s weights are set as trainable. This means that during the training process, all the weights in the model will be updated and optimized, including those in the feature extraction layers. This approach can be useful when training a model from scratch, as it allows the entire model to adapt to the new data and learn the best feature representations for the task at hand.

For the geometric distortion and spatial resolution assessments, these models have binary output indicating the pass or fail of the test. For the low contrast assessment, the models classify the image into 10 classes representing the number of visible spokes.

For all the models, a thorough hyperparameter tuning process was conducted using the Keras Tuner library. The best hyperparameter including, number of filters, the number of units in the densely connected layer, learning rate, the batch size and the number of training epochs were determined through random search with 10 trials.

### Gold standard measurements

For all image quality parameters, two-step process is used to ensure the accuracy of the data, one done by an in-house software and the other by a medical physicist. The software is used to automatically localize the phantom, extract features, and evaluate the quality parameters. The results generated by the software are then reviewed and validated by a medical physicist, who can identify and correct any errors that may have been introduced by the software. These validated results represent the gold standard on which our models are trained.

## Results

### Geometric distortion & spatial resolution

The best hyperparameters obtained for the custom developed CNN model utilized with the geometric distortion were: 96 filters, 256 filters and 192 filters for the first, second and third convolution layers respectively. 128 units for the densely connected layer, 0.001 for the learning rate, 16 for the batch size and 10 training epochs. The best accuracy achieved on the training set was 100% and a precision equal to 100%.

For the spatial resolution, the optimal hyperparameter values for the custom developed CNN model were: 96 filters, 64 filters and 224 filters for the first, second and third convolution layers respectively. 96 units for the densely connected layer, 0.001 for the learning rate, 32 for the batch size and 10 training epochs. The best accuracy achieved on the training set was 100% with a precision equal to 100%.

Table 1 presents the results of the performance evaluation of the geometric distortion and spatial resolution prediction approach using various deep learning architectures, with the transfer learning scenario. The metrics used to evaluate the performance of the approach are the accuracy and the precision. The results on the training set show that when utilizing VGG16 and VGG19, we achieved an accuracy of 99% for the spatial resolution and the geometric distortion tests. For the Resnet50, the accuracy was 100% and 98% for the spatial resolution and the geometric distortion tests. For the InceptionV3 it was 99 and 98%. For the EfficientNetB0, the accuracy was 80% and for the EfficientNetB5 it was 90 and 91%.

**Table 1:** Accuracy and precision of deep learning models using transfer learning for spatial resolution and geometric distortion. All the parameters are frozen

	Spatial resolution		Geometric distortion	
	Accuracy	Precision	Accuracy	Precision
VGG16	0.99	1	0.99	1
VGG19	0.99	1	0.99	1
Resenet50	1	1	0.97	1
InceptionV3	0.99	1	0.98	0.96
EfficinetNetB0	0.8	0.8	0.8	0.46
EfficinetNetB5	0.9	0.97	0.91	0.79

Table 2 presents the results of the performance evaluation of the spatial resolution and geometric distortion prediction approach using various deep learning architectures, when training the models from scratch. The results on the training set show that when utilizing VGG16 and VGG19, we achieved an accuracy of less than 60% for both the spatial resolution and the geometric distortion tests. For the Resnet50, the accuracy was 98% and 90%. For the InceptionV3 it was 99 and 97%. For the EfficientNetB0, the accuracy was 97 and 95% and for the EfficientNetB5, the accuracy was 91 and 93% for the spatial resolution and the geometric distortion.

**Table 2:** Accuracy and precision of deep learning models trained from scratch for spatial resolution and geometric distortion

	Spatial resolution		Geometric distortion	
	Accuracy	Precision	Accuracy	Precision
VGG16	0.46	0.6	0.49	0.6
VGG19	0.55	0.45	0.52	0.63
Resenet50	0.98	0.55	0.9	0.61
InceptionV3	0.99	0.6	0.97	0.7
EfficinetNetB0	0.97	0.57	0.95	0.61
EfficinetNetB5	0.91	0.59	0.93	0.7

The effect of dataset size on model performance was investigated. Upon expanding the dataset and increasing the batch size to 2200 images, notable improvements in accuracy and precision were observed. The VGG16 model achieved an accuracy of 89% and a 92% for both the spatial resolution and the geometric distortion tests. The VGG19 model achieved an accuracy of 94% and 92%. The ResNet50 model achieved an accuracy of 100% and 97%. For the inceptionV3 model, increasing data yielded an accuracy of 100% and 100%. The EfficientNetB0 model achieved an accuracy of 99% and 98%. The EfficientNetB5 model exhibited performance with an accuracy of 96% and 98% for the spatial resolution and the geometric distortion.

### Low contrast

Table 3 summarizes the optimal hyperparameter values for the first CNN model, providing insights into the configuration choices made during model development.

**Table 3:** Best hyperparameter values for the deep learning model used for spatial resolution and geometric distortion

Hyperparameter	Best Value
Number of filters (1st convolution layer)	48
Number of filters (2nd convolution layer)	64
Number of filters (3rd convolution layer)	64
Number of units in the densely connected layer	96
Learning rate	0.00001
Batch size	16
Epochs	20

The best hyperparameters obtained were: 48 filters, 64 filters and 64 filters for the first, second and third convolution layers respectively. 96 units for the densely connected layer, 0.00001 for the learning rate, 16 for the batch size and 20 training epochs.

**Fig. 6:**
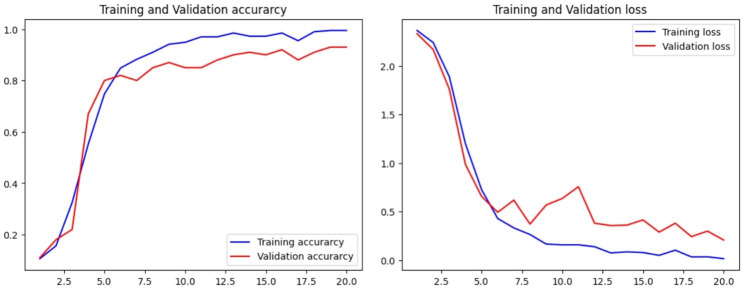
Accuracy and loss graphs for the first CNN model

Figure 6 illustrates the accuracy and loss graphs created during the training and testing, procedures.

After 20 epochs, on the training set the model achieved results with 95% accuracy, 95% recall, approximately 96% precision, and an F1 score of 95%.

Table 4 summarizes the optimal hyperparameter values for the second CNN model, providing insights into the configuration choices made during model development.

**Table 4:** Best hyperparameter values for the deep learning model used for spatial resolution and geometric distortion

Hyperparameter	Best Value
Number of filters (1st convolution layer)	16
Number of filters (2nd convolution layer)	48
Number of filters (3rd convolution layer)	96
Number of filters (4th convolution layer)	384
Number of units in the densely connected layer	64
Learning rate	0.001
Batch size	64
Epochs	30

**Fig. 7:**
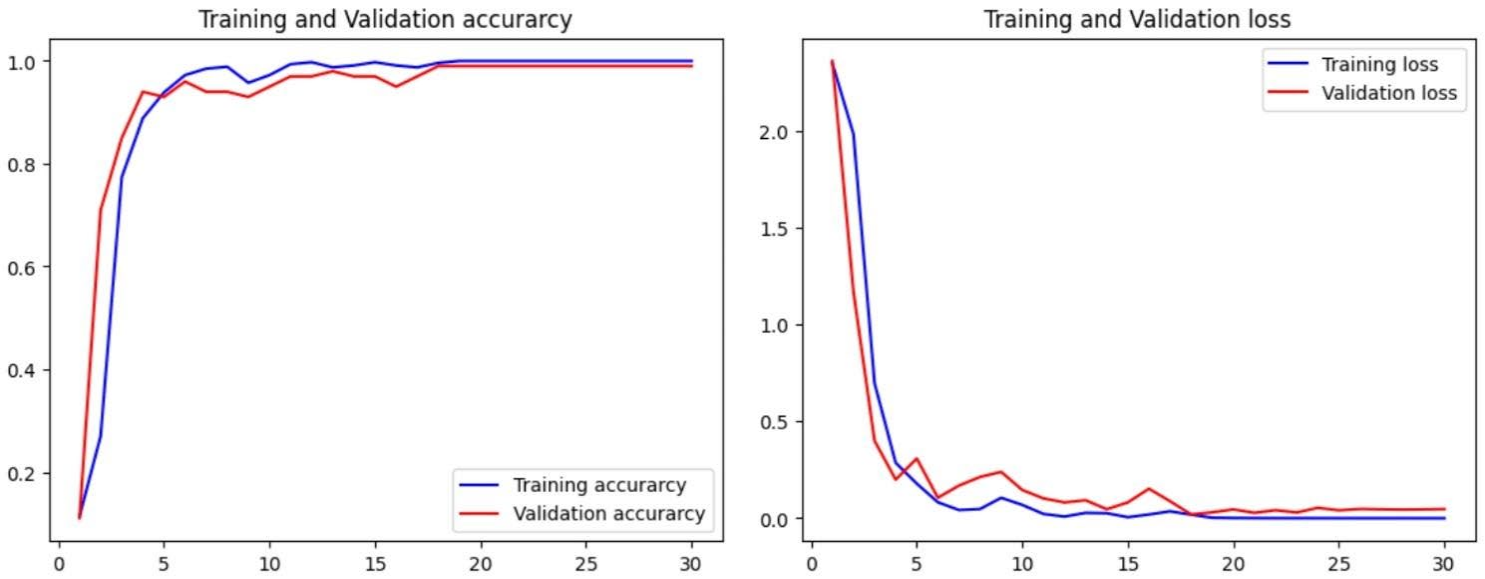
Accuracy and loss graphs for the second CNN model

The findings revealed that best hyperparameters obtained were 16 filters, 48 filters, 96 filters, and 384 filters for the first, second, third and fourth layers respectively. 64 units in the densely connected layer, a learning rate of 0.001, a batch size of 64 and 30 training epochs. Figure 7 shows the accuracy and loss graphs created during the training and testing, procedures.

After 30 epochs, on the training set the model achieved results with 100% accuracy, 100% recall, approximately 100% precision, and an F1 score of 100%.

This section presents the evaluation of predeveloped deep learning models in predicting the number of visible spokes in low-contrast images. The assessment is presented in Table 5, displaying results in both transfer learning and from-scratch scenarios on the training set.

**Table 5:** Accuracy and precision of deep learning models for 10-class classification of MRI images using transfer learning and training from scratch

	Transfer learning		From scratch	
	Accuracy	Precision	Accuracy	Precision
VGG16	31	34	11	10
VGG19	18	17	12	10
Resenet50	16	4	14	11
InceptionV3	71	60	13	10
EfficinetNetB0	58	64	11	11
EfficinetNetB5	74	56	93	92

In the transfer learning scenario, for VGG16, optimal parameters included a learning rate of 0.00001, a batch size of 16, and 15 training epochs. This yielded accuracy, recall, precision, and an F1 score of 11%, 19%, 14%, and 14% respectively. VGG19 achieved 18% accuracy, 18% recall, 17% precision, and a 15% F1 score with parameters of a learning rate of 0.001, a batch size of 16, and 5 training epochs. InceptionV3 showcased an accuracy of 71%, a recall of 63%, precision of 60%, and an F1 score of 58% with a learning rate of 0.001, a batch size of 64, and 20 training epochs. ResNet50 demonstrated 16% accuracy, 14% recall, 4% precision, and a 6% F1 score, utilizing a learning rate of 0.00001, a batch size of 64, and 5 training epochs. EfficientNetB0 achieved 58% accuracy, 58% recall, 64% precision, and a 58% F1 score with parameters of a learning rate of 0.001, a batch size of 32, and 10 training epochs. EfficientNetB5 garnered 74% accuracy, 56% recall, 93% precision, and a 92% F1 score with a learning rate of 0.0001, a batch size of 64, and 20 training epochs.

When training models from scratch, with all layers fine-tuned, distinct behaviors emerged. For VGG16, results included 11% accuracy, 9% recall, 10% precision, and a 10% F1 score, utilizing a learning rate of 0.00001, a batch size of 64, and 15 training epochs. VGG19 achieved 12% accuracy, 8% recall, 10% precision, and a 10% F1 score with parameters of a learning rate of 0.0001, a batch size of 64, and 20 training epochs. ResNet50 demonstrated 14% accuracy, 10% recall, 8% precision, and a 10% F1 score, utilizing a learning rate of 0.00001, a batch size of 32, and 10 training epochs. InceptionV3 showcased 13% accuracy, 13% recall, 10% precision, and an 18% F1 score with a learning rate of 0.001, a batch size of 64, and 15 training epochs. EfficientNetB0 yielded 11% accuracy, 10% recall, 11% precision, and an 11% F1 score, utilizing a learning rate of 0.00001, a batch size of 64, and 10 training epochs. EfficientNetB5 achieved remarkable performance with 93% accuracy, 90% recall, 92% precision, and a 91% F1 score, employing a learning rate of 0.001, a batch size of 16, and 15 training epochs.

Several models experienced challenges in achieving convergence when fine-tuning all layers. To address this, an extensive investigation was undertaken first by modifying the number of fine-tuned layers, and second by increasing the batch size as illustrated in Table 6.

**Table 6:** Accuracy and precision of deep learning models for 10-class classification of MRI images with fine-tuning and training from scratch

	Fine-tuned layers			From scratch (batch size = 3700 images)	
	Number of fine-tuned layers	Accuracy	Precision	Accuracy	Precision
VGG16	5	88	76	73	70
VGG19	6	91	84	71	72
Resenet50	50	10	8	35	35
InceptionV3	50	57	37	21	19
EfficinetNetB0	50	49	56	19	18
EfficinetNetB5	50	100	100	90	88

The VGG16 model achieved optimal results by fine-tuning 5 layers with an accuracy of 88% and a precision of 76%. Similarly, for the VGG19 model, fine-tuning 6 layers yielded peak performance, achieving an accuracy of 91% and a precision of 84%.

In contrast, the ResNet50 model struggled to achieve convergence even after experimenting with different fine-tuning layer configurations. Conversely, for the inceptionV3 model, fine-tuning 50 layers proved optimal, yielding an accuracy of 57% and a precision of 37%. The EfficientNetB0 model, by fine-tuning 50 layers achieved an accuracy of 49% and a precision of 56%. Remarkably, the EfficientNetB5 model exhibited exceptional performance by fine-tuning 50 layers with an accuracy and precision of 100%.

After increasing the batch size to 3700 images, The VGG16 model achieved an accuracy of 73% and a precision of 70%. The VGG19 model achieved an accuracy of 71% and a precision of 72%. The ResNet50 model achieved an accuracy of 35% and a precision of 35%. For the inceptionV3 model, increasing data yielded an accuracy of 21% and a precision of 19%. The EfficientNetB0 model achieved an accuracy of 19% and a precision of 18%. The EfficientNetB5 model exhibited performance with an accuracy and precision of 90% and 88% respectively.

## Discussion

The versatility of MRI in terms of the myriad of possible imaging sequences and potential examinations is one of its primary advantages, but this versatility poses a challenge for quality assurance along with quality control procedures.

The ACR accreditation recommendations are widely accepted as international best practice in MRI QC. Manual tests of the ACR parameters consist of multiple labor-intensive measurements (slice thickness, slice location, contrast, and geometric distortion). Each of these measurements can consume an average of 5 min. Furthermore, results obtained depend on user defined windowing levels and the guidance definitions of leveling and windowing are approximate and subjective. In response to these challenges, the application of artificial intelligence approaches emerges as a promising area of research in the field of medical image analysis.

The objective of this study was to develop and evaluate image-based deep learning-based approaches for the assessment of geometric distortion and spatial resolution in Magnetic Resonance (MR) images. The optimization of hyperparameters for custom Convolutional Neural Network (CNN) models, as well as the analysis of popular pre-trained models including VGG16, VGG19, EfficientNetB0, EfficientNetB5, Resnet50, and InceptionV3, yielded valuable insights into their efficacy for the specified tasks.

The first custom-developed CNN model achieved perfect accuracy and precision scores for geometric distortion and spatial resolution assessment. These tasks are relatively simple compared to low contrast assessment.

Pre-trained models using transfer learning performed well on both tasks, while training from scratch yielded varying results. Some models, such as ResNet50 and InceptionV3, maintained competitive performance, while others, such as VGG16 and VGG19, struggled to achieve accuracy values above 60%.

Increasing the data size from 600 to 2200 led to convergence of all models with improved prediction results. This suggests that to use existing models for geometric distortion and spatial resolution assessment, we can either use transfer learning or train from scratch with a large dataset.

Transforming the images from Cartesian to polar coordinates was the most important step in improving the accuracy of all models for low contrast image quality assessment. This is because polar image transforms can separate the segmentation task from the localization task, allowing the neural network to focus on learning to segment the objects without having to worry about where they are located in the image.

The custom-developed CNN models performed well, with the first model achieving strong accuracy, recall, precision, and F1 scores, and the second model achieving perfect scores across all metrics after 30 training epochs.

The pre-trained models performed variably when predicting the number of visible spokes in low contrast images. When trained from scratch with all layers fine-tuned, only EfficientNetB5 converged and achieved good accuracy. The other models, VGG16, VGG19, ResNet50, InceptionV3, and EfficientNetB0, failed to converge.

However, when some layers were frozen and others were fine-tuned, all models performed better. Additionally, increasing the batch size from 1000 to 3700 images led to convergence of all models with improved prediction results. These findings suggest that transfer learning is preferable when the batch size is small, while training from scratch is effective with a large batch size. Additionally, with a small batch size, we can fine-tune some layers instead of all layers.

This study represents the first attempt to evaluate the potential of image-based deep learning for automatic ACR quality control. Our approach differs from previous studies [11, 12], which utilized machine learning and deep learning techniques but applied on manually created characteristics which is labor-intensive and increases error rates. Our approach, on the other hand, utilizes image-based deep learning models to directly analyze MR images. This allows for a more comprehensive analysis of the images, as compared to the feature-based methods used in previous studies.

Future investigations could focus on refining and optimizing the training process for models that exhibit challenges when trained from scratch. Techniques such as curriculum learning, progressive training, and ensemble approaches could potentially enhance the convergence and performance of these models. Additionally, exploring the combination of transfer learning and fine-tuning strategies could leverage the strengths of pre-trained models while adapting them to specific medical imaging tasks. This approach may further improve the generalization and robustness of the models. Furthermore, the incorporation of domain-specific features and considerations into the model architecture could enhance the models’ understanding of medical image characteristics and potentially lead to more accurate predictions.

In summary, the study’s findings contribute to our understanding of utilizing deep learning models for MR quality assessment. The combined insights from custom-developed models and pre-trained architectures offer a versatile approach to address various challenges in geometric distortion, spatial resolution, and low contrast image quality assessment. These findings underscore the potential of deep learning techniques to revolutionize medical image analysis and contribute to advancements in patient care and diagnosis.

## Coclusions

In this study, we undertook a comprehensive exploration of deep learning models for the assessment of geometric distortion, spatial resolution, and low contrast image quality in MRI images.

Deep learning models demonstrated promising potential for the assessment of geometric distortion, spatial resolution, and low contrast image quality in MRI. Custom CNN models achieved superior performance on all tasks, while pre-trained models showed promise for geometric distortion and spatial resolution assessment. The findings highlight the potential of deep learning to revolutionize MRI quality assessment.

## Data Availability

The datasets used and/or analysed during the current study are available from the corresponding author on reasonable request.
